# Dectin-1 Facilitates IL-18 Production for the Generation of Protective Antibodies Against *Candida albicans*

**DOI:** 10.3389/fmicb.2020.01648

**Published:** 2020-07-16

**Authors:** Hui Shen, Yuetian Yu, Si-Min Chen, Juan-Juan Sun, Wei Fang, Shi-Yu Guo, Wei-Tong Hou, Xi-Ran Qiu, Yu Zhang, Yuan-Li Chen, Yi-Da Wang, Xin-Yu Hu, Liangjing Lu, Yuan-Ying Jiang, Zui Zou, Mao-Mao An

**Affiliations:** ^1^Department of Pharmacology, Shanghai Tenth People’s Hospital, Tongji University School of Medicine, Shanghai, China; ^2^Department of Laboratory Diagnosis, Shanghai East Hospital, Tongji University School of Medicine, Shanghai, China; ^3^Department of Rheumatology, Renji Hospital, School of Medicine, Shanghai Jiao Tong University, Shanghai, China; ^4^Department of Critical Care Medicine, Renji Hospital, School of Medicine, Shanghai Jiao Tong University, Shanghai, China; ^5^Department of Anesthesiology, Changzheng Hospital, Second Military Medical University, Shanghai, China

**Keywords:** *C. albicans*, invasive candidiasis, vaccine, antibody, dectin-1

## Abstract

Invasive candidiasis (IC) is one of the leading causes of death among immunocompromised patients. Because of limited effective therapy treatment options, prevention of IC through vaccine is an appealing strategy. However, how to induce the generation of direct candidacidal antibodies in host remains unclear. *Gpi7* mutant *C. albicans* is an avirulent strain that exposes cell wall β-(1,3)-glucans. Here, we found that vaccination with the *gpi7* mutant strain could protect mice against invasive candidiasis caused by *C. albicans* and non-*albicans Candida* spp. The protective effects induced by *gpi7* mutant relied on long-lived plasma cells (LLPCs) secreting protective antibodies against *C. albicans*. Clinically, we verified a similar profile of IgG antibodies in the serum samples from patients recovering from IC to those from *gpi7* mutant-vaccinated mice. Mechanistically, we found cell wall β-(1,3)-glucan of *gpi7* mutant facilitated Dectin-1 receptor dependent nuclear translocation of non-canonical NF-κB subunit RelB in macrophages and subsequent IL-18 secretion, which primed protective antibodies generation *in vivo*. Together, our study demonstrate that Dectin-1 engagement could trigger RelB activation to prime IL-18 expression and established a new paradigm for consideration of the link between Dectin-1 mediated innate immune response and adaptive humoral immunity, suggesting a previously unknown active vaccination strategy against *Candida* spp. infection.

## Introduction

Invasive candidiasis (IC) is one of leading death causes among hospitalized individuals, especially immunocompromised patients (Kullberg and Arendrup, 2015; McCarty and Pappas, 2016). It is the fourth most common nosocomial bloodstream infection with a mortality rate about 40–50% (Arendrup, 2010; Yang et al., 2017). Anticandidal therapy is often of limited effectiveness in these patients, resulting in high rates of mortality and morbidity (Arendrup, 2010; Kullberg and Arendrup, 2015; McCarty and Pappas, 2016; Yang et al., 2017). The diagnosis of IC is not always possible due to difficulties in isolating the *Candida* cells from clinical samples, but clearly defined risk factors, such as immunocompromise, have been established for IC (Clancy and Nguyen, 2013; Candel et al., 2017). Therefore, the prevention of IC through a proven, effective vaccine is an appealing strategy.

Although recent studies have highlighted the crucial roles of T_H_1 and T_H_17 cell-mediated immunity in host defense against infection caused *Candida* spp., both protective immune serum and antibodies have shown remarkable efficacy (Bugli et al., 2013; Drummond et al., 2014; Verma et al., 2014). These findings have special relevance for vaccination, especially in partly or totally immunocompromised individuals. In general, vaccination can induce antibody generation in at-risk patients before they become immunocompromised. In addition, because of the longevity (weeks to months depending on the IgG subclass), these IgG antibodies might persist at a protective titer even during prolonged immunosuppression. However, strategies for directly inducing candidacidal antibody generation in the host have not been developed.

Dectin-1, a spleen tyrosine kinase (Syk)-coupled C-type lectin receptors (CLR) expressed on myeloid cells, recognizes β-(1,3)-glucan on cell surface of various fungi (Taylor et al., 2007). Dectin-1 engagement by β-(1,3)-glucan induces phosphorylation of the immunoreceptor tyrosine-based activation motif-like sequence within the cytoplasmic domain of Dectin-1 (Rogers et al., 2005; Underhill et al., 2005). The subsequent phosphorylation of Syk induces the assembly of caspase recruitment domain 9 (CARD9) protein, the adaptor proteins Bcl-10 and MALT1 (Rogers et al., 2005; Underhill et al., 2005; Gross et al., 2006). The CARD9-Bcl-10-MALT1 scaffold contributes to NF-kB pathway activation and thus helps prime the T helper (T_H_) cell immune response (Gross et al., 2006; Gringhuis et al., 2009; Drummond et al., 2014; Verma et al., 2014; Xu et al., 2018). Dectin-1 mediated T_H_1 and T_H_17 cell immune responses are effective in host defense against fungal infection (Verma et al., 2014). Previous studies suggested that Dectin-1 could prime T_H_2 cell response by inducing non-canonical NF-kB subunit RelB activation (Gringhuis et al., 2009; Xu et al., 2018). However, little is known about whether T_H_2 cell response mediated by Dectin-1 can trigger humoral immunity and protective antibodies production in host.

*Candida albicans* is the most common *Candida* spp. that can cause invasive infection (Arendrup, 2010; Kullberg and Arendrup, 2015; McCarty and Pappas, 2016; Yang et al., 2017). *C. albicans* shields surface β-(1,3)-glucan, except in the region between the mature bud and parent yeast cell, for evading host Dectin-1 binding (Gantner et al., 2005; Wheeler et al., 2008). Most cell wall proteins (CWPs) of *C. albicans* are glycosylphosphatidylinositol (GPI)-anchored proteins (GPI-CWPs), which are linked to cell wall β-(1,6)-glucan through a GPI remnant (Richard and Plaine, 2007; Chaffin, 2008). *C. albicans GPI7* is responsible for adding ethanolaminephosphate to the second mannose in GPI anchor biosynthesis and key for the linkage of GPI-anchored protein to the cell wall (Richard et al., 2002; Imhof et al., 2004; Richard and Plaine, 2007). In our previous study, we generated an avirulent *C. albicans* strain (*gpi7* mutant) lacking GPI-CWPs and demonstrated that the surface β-(1,3)-glucan of *gpi7* mutant was exposed (Shen et al., 2015). The findings encouraged us to investigate whether vaccination with this avirulent mutant could confer protection against infection caused by wild type *C. albicans*.

This study found that the *gpi7* mutant *C. albicans* with surface β-(1,3)-glucan exposure could induce hosts to generate protective antibodies against IC in mice and demonstrated that IL-18 plays a central role in the type 2 response contributing to this immunoprotection. The mechanistic analysis revealed that Dectin-1 engagement by surface β-(1,3)-glucan of the *gpi7* mutant could facilitate activation of the non-canonical NF-kB subunit RelB, and this activation regulates IL-18 expression to prime the type 2 response. Clinically, we verified a similar profile of IgG antibodies in serum samples from patients recovering from IC to those of *gpi7* mutant-vaccinated mice.

## Materials and Methods

### Reagents, Antibodies and Plasmids

Coomassie brilliant blue, IPTG and DTT were purchased from Sangon Biotech. The p65 inhibitor helenalin was purchased from Abcam. RelB inhibitor 1,25(OH)_2_D_3_ and HF-pyridine was purchased from Merck. Zymosan were purchased from sigma. Ni-nitrilotriacetic acid (Ni-NTA) were purchased from QIAGEN. V450-conjugated anti-B220 antibody (Clone RA3-6B2, BioLegend), Phycoerythrin-anti-CD44 antibody (Clone MEM-85, BioLegend), FITC-conjugated anti-MHCII antibody (Clone M5/114.15.2, BioLegend), Allophycocyanin-conjugated anti-CD138 antibody (Clone 281-2, BioLegend), Allophycocyanin-conjugated anti-CD80 antibody (Clone 16-10A1, BioLegend), FITC-conjugated anti-CD273 antibody (Clone 122, Invitrogen), Phycoerythrin-conjugated-CD73 antibody (Clone TY/11.8, BioLegend), Anti-mouse IL-4 Antibody (Cat# ab93503, Abcam), and Alexa-488-labeled goat anti-mouse antibody (Cat# A11059, Life Technologies) were used in flow cytometry (isotype antibodies were used as controls). The neutralizing anti-CD4 antibody was purchased from Invitrogen. And the neutralizing anti-IL-17A antibody and anti-IFN-γ antibody were purchased from BioLegend. The neutralizing anti–IL-18 antibody was purchased from R&D Systems. Antibodies against RelB and PCNA were obtained from Cell Signaling Technologies. pET-21a (+) was obtained from Novagen.

### Mice

Female C57BL/6 and BALB/c background athymic nude mice were obtained from Shanghai Laboratory Animal Center (SLAC) of the Chinese Academy of Sciences (Shanghai, China). Dectin-1-deficient (Dectin-1^–/–^) mice were kindly provided by Dr. Gordon D. Brown (the mice were backcrossed for nine generations on the C57BL/6 background).

### Strain Growth Conditions

*gpi7* mutant *C. albicans* was generated in our previous study (Shen et al., 2015; Zhang et al., 2016). *C.albicans* SN152 was kindly provided by Dr. Noble SM (University of California-San Francisco). *C. albicans* SC5314 was kindly provided by Sanglard D (Centre Hospitalier Universitaire Vaudois). The clinical isolate *C. albicans* 0304103 was kindly provided by Dr. Jun Gu (Changhai Hospital, Shanghai, China), and *ssa1* mutant *C. albicans* was kindly provided by Dr. Filler SG (State University of New York) (Sun et al., 2010). *C. glabrata* ATCC28226, *C. parapsilosis* ATCC 22019, *C. krusei* ATCC 6258 and *C. tropicalis* ATCC 750 were obtained from ATCC.

Sabouraud dextrose agar (SDA) plates (4% dextrose,1% peptone, and 1.8% agar) were used for isolation of individual *Candida* clones. Individual *Candida* clones were cultured in yeast peptone dextrose (YPD) liquid medium (2% dextrose, 2% peptone, and 1% yeast extract) with shaking incubator at 30°C. For *C. albicans* heat-inactivation, *C. albicans* SN152 (2.5 × 10^7^/ml) were boiled in PBS for 10 min. BL21 (DE3) pLysS was purchased from Tiangen Biotech, and cultured on LB plates (1%NaCl, 1% tryptone, 0.5% yeast extract, 1.5% Agar) for isolation of individual clones and cultured in LB liquid medium at 37°C in a shaking incubator for *C. albicans* proteins expression.

### Serum Samples and Ethics Statement

Ten serum samples were collected from recovering IC patients, and 10 serum samples were taken from deceased IC patients with clinical and microbiological evidence in intensive care unit (ICU) of Shanghai Tenth People’s Hospital and Changzheng Hospital (Shanghai, China) and were saved at −80°C. The patients for serum samples collecting were age 50–70 years old, and the neutropenia patients (absolute neutrophil count <500 cells/mm^3^) were excluded from our study. The iatrogenic risk factors of the included IC patients included broad-spectrum antibiotics (5, 25%), central venous catheters (4, 20%), parenteral nutrition (6, 30%), and abdominal or thoracic surgery (5, 25%). This study was approved by the Review Board and Ethics Committee (Permit number: 2018-Clinical-Res-172).

### Murine Invasive Candidiasis Model

For invasive *Candida* spp. infection *in vivo*, C57BL/6 or athymic nude (BALB/c background) female mice were intravenously infected with 200 μl of live *Candida* cells in sterile saline. The mice were daily monitored for survival or sacrificed at day 2 post-infection for fungal burden assessment and pathological examination. The livers and kidneys were collected and fixed in neutral formalin (10%) for H&E and PAS staining or homogenized in 0.5 ml of PBS for fungal burden assay. Homogenate supernatants of kidney were collected and stored at −80°C until cytokines measurements.

### Vaccination and Candida spp. Reinfection Models

C57BL/6 female mice, 6–8 weeks old, were intravenously vaccinated with live *gpi7 mutant* (5 × 10^5^ CFU per mouse) at day 1 and day 14, and re-infected with wild-type *Candida* spp. at day 28. UV-inactive parental strain *C. albicans* SN152-vaccination was used as control. To investigate the role of T_H_1 and T_H_17 mediated immune response in *gpi7* mutant induced protective effect against *Candida* spp. infection, *gpi7* mutant-vaccinated mice were intravenously injected with anti-IFN-γ antibody (clone XMG1.2, 500 μg per mouse) or anti-IL-17A antibody (clone TC11-18H10.1, 100 μg per mouse).

### Cytokines Measurement

Concentrations of TNF-α, IL-6, IL-4, IL-13, and IL-18 in spleen and serum homogenates and concentrations of IL-17 and IFN-γ in kidney homogenates were assayed under manufacturer’s instructions by Ready-Set-Go cytokine kits (Invitrogen) in triplicates.

### Flow Cytometry Analysis

The spleen and bone marrow of mice were homogenized in 1 ml PBS and were subsequently passed through a 70-mm filter. For leukocytes isolation, the mixture cells were centrifuged in a 40%/70% percoll gradient (Huang et al., 2016). Cells were counted and blocked with PBS containing 5% heat-inactivated FBS and 1 mM sodium azide at 4°C. The cells were then incubated with V450-conjugated anti-B220, phycoerythrin-anti-CD44, allophycocyanin-conjugated anti-CD138 and FITC-conjugated anti-MHCII antibodies to detect LLPCs or V450-conjugated anti-B220, FITC-conjugated anti-CD273 antibodies, allophycocyanin-conjugated anti-CD80 and phycoerythrin-conjugated-CD73 antibodies to detect B_mem_ in a binding buffer at room temperature for 20 min. After being fixed with 1% formaldehyde in PBS, a flow cytometry (BD FACSVerse) were used to evaluate LLPCs and B_mem_ cells.

### Confocal Laser Scanning Microscopy

To analyze antibodies in *gpi7* mutant-induced antiserum that bind to epitopes on the surface of *C. albicans*, exponentially growing *C. albicans* SC5314 yeast cells were incubated with serum samples from *gpi7* mutant-vaccinated mice at 4°C overnight and then stained with alexa-488-labeled goat anti-mouse secondary antibodies (Life Technologies) for 1 h at 30°C. A confocal laser scanning microscope (TCS SP5; Leica) were used to scanned the stained cells (63 × magnification). LAS AF Lite program was used to acquire and analysis micrograph images.

### Identification of Immunogenic Proteins on Cell Wall of *Gpi7* Mutant

Covalent and non-covalent *C. albicans* CWPs were isolated according to previously published methods (Zhang et al., 2016). The CWPs of *C. albicans* mainly included GPI-APs, non-covalent CWPs and Pir proteins (proteins with internal repeats). The cell wall debris were treated by undiluted HF-pyridine at 0°C for 3 h for GPI-CWPs releasing. Then the cell wall debris were treated by 30 mM NaOH at 4°C for 16 h for Pir proteins specifically releasing with. The non-covalent CWPs of *C. albicans* were extracted in an SDS buffer (10 mM DTT, 100 mM EDTA, 2% SDS, 50 mM Tris–HCl, and; pH 8.0). All the CWPs were then mixed for a subsequent experiment.

To determine whether *gpi7*-induced antiserum can specifically detect proteins from *C. albicans*, the whole CWPs mixture from wild-type *C. albicans* was fracted by SDS-PAGE and then subjected to immunoblotting analysis using *gpi7*-induced antiserum or wild-type *C. albicans* SN152-induced serum. Total CWPs from wild-type *C. albicans* stained with Coomassie brilliant blue on SDS-PAGE was used as a control. Then, the gel bands of different proteins were isolated from the control lane for LC-MS/MS analysis by high-resolution instruments (LTQ-Orbitrap XL and Velos, Thermo Fisher Scientific). MaxQuant software was used for peptide/protein identification from the raw files.

### Expression and Purification of Cell Wall-Localized Moonlighting Proteins of *C. albicans*

The genes encoding cell wall-localized moonlighting proteins were amplified from *C. albicans* SC5314 genomic DNA (the primers were listed in Supplementary Table S1) and cloned into pET-21a (+), which includes a 6 × His tag. The plasmid was then transformed into BL21 (DE3) pLysS cells to express His-fused moonlighting proteins. The transformants were cultured at 37°C for 12 h and diluted in fresh LB medium (1:100). Once the optical density at 600 nm (OD600) of the medium reached 0.6, the cells were grown for 16 h at 16°C in LB medium containing 0.1 mM IPTG. The Ni-nitrilotriacetic acid (Ni-NTA) (QIAGEN) were used to purified the His-fused moonlighting proteins.

### Determination of the IgG Titers in Antiserum

After coating 96-well microtiter plates with a recombinant *C. albicans* moonlighting protein (0.1 μg per well), the IgG titers in serum samples from *gpi7* mutant-vaccinated mice or IC patients were determined by indirect ELISA. The relative amounts of IgG are shown in a heat map produced using Heml 1.0.3.3 (heatmap illustrator).

### Thioglycollate-Elicited Peritoneal Neutrophils and Macrophages Preparation

Thioglycollate-elicited peritoneal neutrophils and macrophages were harvested as previously published method (Taylor et al., 2007). Thioglycollate (Merck, 3%, wt/vol) were injected into the abdominal cavity of C57BL/6 mice (2 ml per mouse). Peritoneal cells were harvested through washing with PBS containing 0.5 mM EDTA 14 h later for neutrophils or 72 h later macrophages collection. The neutrophils and macrophages were cultured in RPMI1640 containing FBS (10%, vol/vol).

### Serum Opsonophagocytic Phagocytosis and Killing Assay

For phagocytosis assay, *C. albicans* SC5314 was incubated with 1 × 10^6^ peritoneal macrophages (MOI = 0.4) accompanied with diluted *gpi7*-induced antiserum at 37°C for 1 h. After washed with PBS, unbound *C. albicans* SC5314 cells were removed and the mixture was plate on SDA agar at 30°C for live *C. albicans* counted after 48 h incubation.

For neutrophils killing assay, *C. albicans* SC5314 (1 × 10^4^ CFU) were incubated with diluted *gpi7*-induced antiserum for 1 h at 30°C. The cells of *C. albicans* SC5314 were collected and incubated with thioglycolate-elicited peritoneal neutrophils at MOI = 0.05 for 1 h at 4°C to make the cells settle, and then transferred to 37°C for further 1 h for killing test. Control plates were placed at 4°C in parallel during the incubation, then the mixture was plated on SDA agar at 30°C for 48 h and the live *C. albicans* were counted.

### Quantitative Real-Time RT-PCR

Total RNA was isolated using TRIzol (Invitrogen) and reverse transcribed using SuperScript III (Invitrogen). Power SYBR Green PCR Master Mix (TaKaRa, RR420A) was then used for quantitative real-time PCR. The amounts of transcript were normalized to those for Gapdh. The primers used in this assay are listed in Supplementary Table S2.

### Immunoblotting Assays

Total cell lysates were extracted by (250 mM NaCl, 1 mM EDTA, 1% NP-40, 50 mM HEPES, protease inhibitors, pH 7.4). Nuclear extracts were extracted by lysis buffer (0.1 mM EDTA, 10 mM HEPES, 10 mM KCl, 0.4% NP-40, protease inhibitors, pH 7.9). Nuclear extracts or total cell lysates were subjected to SDS-PAGE, blotted with the indicated primary antibodies and secondary antibodies, and then developed using an ECL detection system (GE Healthcare).

### Quantification and Statistical Analysis

All experiments performed three biological replicates unless otherwise indicated. Log-rank test was used for survival data analysis. For parametric data, two groups were analyzed by two-tailed Student’s *t*-test and multiple groups were analyzed by one-way analysis of variance (ANOVA). For non-parametric data, the non-parametric *t*-test or ANOVA was used. Statistical significance was set at a *P*-value of 0.05, 0.01 or 0.001.

## Results

### *gpi7* Mutant-Vaccination Protects Mice From IC

**FIGURE 1:**
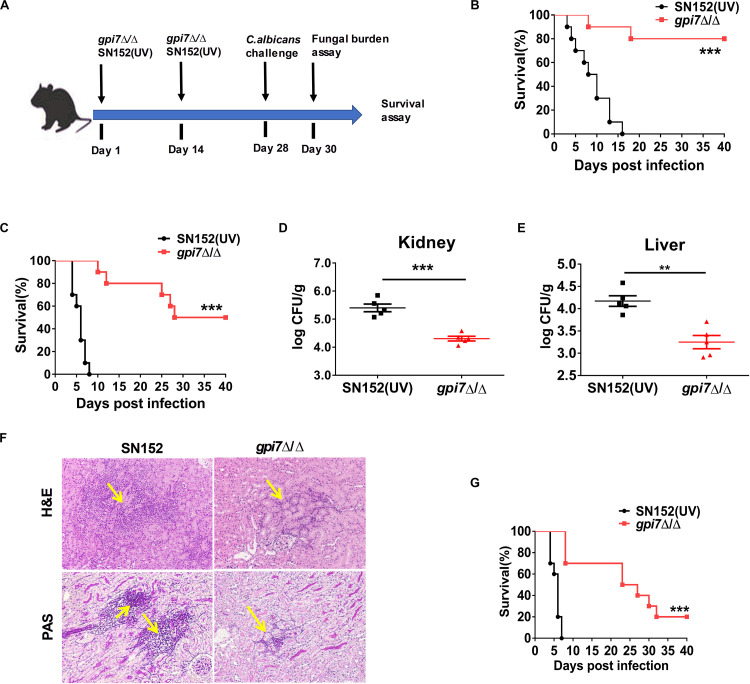
G*pi7* mutant-vaccination protects mice from invasive *C. albicans* infection. **(A)** The strategy for *gpi7* mutant-vaccination and wild-type *C. albicans* reinfection. C57BL/6 mice were intravenously vaccinated with live *gpi7 mutant* (5 × 10^5^ CFU per mouse) at day 1 and day 14, and re-infected with wild-type *C. albicans* at day 28. UV-inactive parental strain *C. albicans* SN152 vaccination was used as control. The kidney and liver fungal burden was determined at day 2 post-infection and survival was surveyed for 40 days’ post-infection. **(B,C)** The survival of *gpi7* mutant-vaccinated C57BL/6 mice were monitored for 40 days’ post systemically infected with *C. albicans* SC5314 (B, 5 × 10^5^ CFU per mouse; C, 1 × 10^6^ CFU per mouse) (*n* = 10 per group). Data are representative of three independent experiments. **(D,E)** The kidney fungal burden **(D)** and liver fungal burden **(E)** of *gpi7* mutant-vaccinated C57BL/6 mice at day 2 post systemically infected with *C. albicans* SC5314 (5 × 10^5^ CFU per mouse) (*n* = 5 per group). Data are representative of three independent experiments. **(F)** Representative H&E (for the inflammatory cells influx and the extent of tissue necrosis) and PAS (for *C. albicans*) staining of kidneys from *gpi7* mutant-vaccinated C57BL/6 mice at day 2 post systemically infected with *C. albicans* SC5314 (5 × 10^5^ CFU per mouse). Arrows indicate inflammatory cells influx and tissue necrosis (H&E staining), and *C. albicans* filaments in the tissues (PAS staining). Magnification = 200 ×. **(G)** Survival of *gpi7* mutant-vaccinated C57BL/6 mice were monitored for over 40 days post systemically infected with clinical isolated *C. albicans* 0304103 (1 × 10^6^ CFU per mouse) (*n* = 10 per group). Data are representative of three independent experiments. **P < 0.01; ***P < 0.001 [Log-rank test **(B,C,G)** and non-parametric *t*-test **(D,E)**].

In previous studies, we constructed *C. albicans gpi7* mutant of which cell surface β-(1,3)-glucan was exposed (Shen et al., 2015). A series of vaccination experiments were performed according to the strategies shown in Figure 1A. *Gpi7* mutant-vaccinated mice or parental strain SN152 (UV-inactivated)-vaccinated mice were subsequently systemically infected with a lethal dose of *C. albicans* SC5314 (Figures 1B–F) or clinical isolate *C. albicans* 0304103 (Figure 1G). When challenged with a lethal dose of *C. albicans* SC5314, the *gpi7* mutant-vaccinated mice presented a markedly higher survival rate than those vaccinated with the parental strain SN152 (Figures 1B,C). Over a 40-day observation period, two *gpi7* mutant-vaccinated mice infected by 5 × 10^5^ CFU *C. albicans* SC5314 died (Figure 1B), and half of the *gpi7* mutant-vaccinated mice infected by 1 × 10^6^ CFU *C. albicans* SC5314 died (Figure 1C). In contrast, all the mice vaccinated with the parental strain SN152 and challenged with *C. albicans* SC5314 died within 20 days (Figures 1B,C). At day 2 and 7 post-infection, *gpi7* mutant-vaccinated mice had significantly lower kidney and liver fungal burdens than those vaccinated with parental strain SN152 (Figures 1D,E and Supplementary Figure S1). Moreover, hematoxylin and eosin (H&E) staining identified fewer inflammatory cell influx and ameliorated tissue necrosis in the kidneys in the *gpi7* mutant-vaccinated mice (Figure 1F, top panel). Periodic acid-Schiff (PAS) staining also revealed fewer hyphae in the kidneys of the *gpi7* mutant-vaccinated mice than in those of the parental strain SN152-vaccinated mice (Figure 1F, bottom panel). In terms of systemically infection by the clinically isolated *C. albicans* 0304103 strain which is more virulent than *C. albicans* SC5314, *gpi7* mutant-vaccination could significantly extent the median survival time of mice, while did not significantly improve the long-term survival (Figure 1G). This result suggested that the level of protection elicited by *gpi7* mutant-vaccination is strain-dependent (mainly related with the pathogenicity of the infected strain).

**FIGURE 2:**
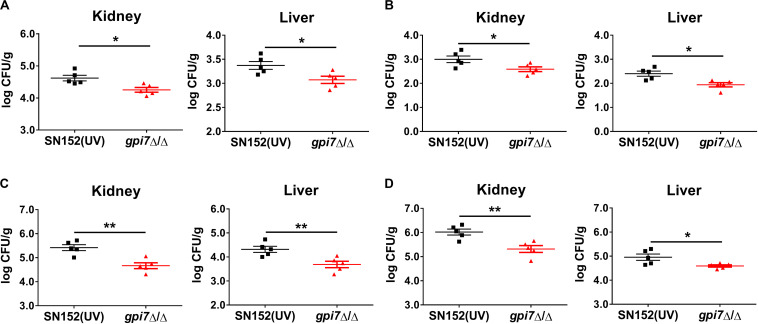
G*pi7* mutant-vaccination protects mice from invasive infections caused by non-*albicans Candida* spp. *Gpi7* mutant-vaccinated C57BL/6 mice were systemically infected with non-*albicans Candida* spp. (*n* = 5 per group). And the kidney fungal burden was determined at day 2 post-infection. **(A)** Kidney and liver fungal burden of *gpi7* mutant-vaccinated mice infected with *C. glabrata* ATCC 28226 (1 × 10^7^ CFU per mouse). **(B)** Kidney and liver fungal burden of *gpi7* mutant-vaccinated mice infected with *C. krusei* ATCC 6258 (1 × 10^7^ CFU per mouse). **(C)** Kidney and liver fungal burden of *gpi7* mutant-vaccinated mice infected with *C. parapsilosis* ATCC 22019 (1 × 10^6^ CFU per mouse). **(D)** Kidney and liver fungal burden of *gpi7* mutant-vaccinated mice infected with *C. tropicalis* ATCC 750 (1 × 10^6^ cells per mouse). Data are representative of three independent experiments. *P < 0.05; **P < 0.01 (Non-parametric *t*-test).

Although *C. albicans* is the most common *Candida* spp., there has been an increase of infections caused by non-*albicans Candida* spp. (Parmeland et al., 2013; Fu et al., 2017). Therefore, *gpi7* mutant-vaccinated mice were also subsequently systemically infected with non-*albicans Candida* spp., including *Candida glabrata*, *Candida krusei*, *Candida parapsilosis* and *Candida tropicalis*. At day 2 post-infection, the *gpi7* mutant-vaccinated mice had significantly lower fungal burdens in the kidneys than those mice vaccinated with parental strain SN152, which indicated that the *gpi7* mutant provided markedly improved immunoprotection (Figure 2).

Furthermore, we examined whether prior exposure to another avirulent *ssa1* mutant without cell surface β-(1,3)-glucan exposure (Sun et al., 2010) could confer protection to infection caused by wild type *C.albicans*. However, after challenge with *C. albicans* SC5314, the fungal burdens in the kidneys and liver of *ssa1* mutant-vaccinated mice were similar to those of mice vaccinated with its complemental strain (Supplementary Figure S2). These results demonstrated that cell surface β-(1,3)-glucan exposure is critical for *C. albicans* induced host immunoprotection.

### LLPC Mediated Humoral Immunity Is Involved in the Immunoprotection Induced by *Gpi7* Mutant

**FIGURE 3:**
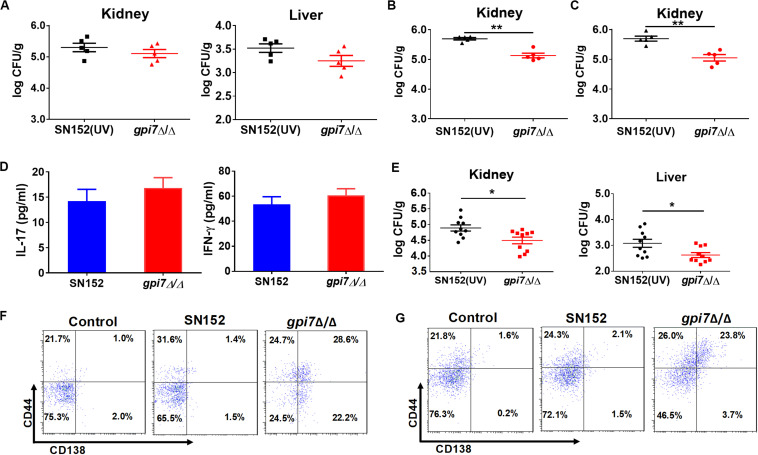
LLPCs mediated humoral immunity is involved in the immunoprotection induced by *gpi7* mutant. **(A)** *Gpi7* mutant-vaccinated athymic nude mice were systemically infected with *C. albicans* SC5314 (5 × 10^5^ CFU per mouse, *n* = 5 per group). And the kidney and liver fungal burdens were determined at day 2 post-infection. Data are representative of three independent experiments. **(B,C)** *Gpi7* mutant-vaccinated C57BL/6 mice treated with anti-IFN-γ antibody (500 μg per mouse) **(B)** or of anti-IL-17A (100 μg per mouse) **(C)** were systemically infected with *C. albicans* SC5314 (5 × 10^5^ CFU per mouse, *n* = 5 per group). And the kidney burden was determined at day 2 post-infection. Data are representative of three independent experiments. **(D)** ELISA assays for IL-17 and IFN-γ in homogenized kidneys from *gpi7* mutant-vaccinated C57BL/6 mice at day 2 post systemically infected with *C. albicans* SC5314 (*n* = 8 per group). Data are means ± SD and are representative of three independent experiments. **(E)** Two hours before systemically infected with *C. albicans* SC5314 (5 × 10^5^ cells per mouse), C57BL/6 mice were treated by 100 μl *gpi7* mutant-induced antiserum. And the kidney and liver fungal burdens were determined at day 2 post-infection (*n* = 10 per group). Data are means ± SD and are representative of three independent experiments. **(F,G)** Flow cytometry for B220^+^CD44^+^CD138^+^MHCII^–^ LLPCs in the spleen **(F)** and bone marrow **(G)** of *gpi7* mutant-vaccinated C57BL/6 mice. Data are representative images of 5 mice. *P < 0.05; **P < 0.01 (Non-parametric *t*-test).

To determine whether innate or adaptive immunity plays a central role in the protective effect of *gpi7* mutant vaccination, we performed vaccination experiments with athymic nude mice. After infected with a lethal dose of *C. albicans* SC5314, the fungal burdens in the kidney and liver of the *gpi7* mutant-vaccinated athymic nude mice were similar to those of the parental strain SN152-vaccinated mice, which suggested that immunoprotection is dependent on the thymus and that adaptive immunity plays a necessary role in this process (Figure 3A).

CD4^+^ T_H_1 and T_H_17 cell-mediated immunity plays important roles in host defense against fungal infection (Drummond et al., 2014; Verma et al., 2014). Interferon gamma (IFN-γ) and IL-17 are the key cytokines produced by Th1 and Th17 cells, respectively. Therefore, we abolished T_H_1 and T_H_17 cell-mediated immunity in *gpi7* mutant-vaccinated mice by neutralizing anti-IFN-γ and anti-IL-17 antibodies. And the efficacy of anti-IFN-γ antibody and anti-IL-17A antibody were confirmed by detecting serum IFN-γ and IL-17A by ELISA (Supplementary Figure S3).

The results demonstrated that neutralizing anti-IFN-γ antibody (Figure 3B) or anti-IL-17 antibody (Figure 3C) did not affect the immunoprotection against *C. albicans* infection induced by *gpi7* mutant vaccination. Next, T_H_1 and T_H_17 cell mediated immune responses were also evaluated by measuring IFN-γ and IL-17 levels in the kidneys of *gpi7* mutant-vaccinated mice at day 2 post *C. albicans* SC5314 challenge, and no difference was found between the *gpi7* mutant- and parental strain SN152-vaccinated mice (Figure 3D). The above results suggested that T_H_1 and T_H_17 cell-mediated immunity were not involved in the protective effect induced by *gpi7* mutant vaccination.

We subsequently investigated the role of B cell-mediated humoral immunity in the protective effects inducted by *gpi7*-mutant vaccination. An adoptive immunization experiment demonstrated that the serum from *gpi7* mutant-vaccinated mice could protect mice from systemic *C. albicans* infection (Figure 3E). This result suggested that B cell-mediated humoral immunity played a critical role in *gpi7* mutant induced protective effect. Memory in humoral immunity depends on the differentiation and maintenance of memory B cells (B_mem_) or LLPCs. Our results revealed that the number of B220^+^CD44^+^CD138^+^MHCII^–^ LLPCs in the bone marrow (Figure 3F) and spleen (Figure 3G) were higher in the *gpi7* mutant-vaccinated mice than in the parental strain SN152-vaccinated mice. However, no difference in B220^+^ CD273^+^CD80^+^CD73^+^ B_mem_ cells were found between the *gpi7* mutant- and parental strain SN152-vaccinated mice (Supplementary Figure S4). These results suggested that maintenance of the protective effect against infection induced by *gpi7* mutant vaccination can be mainly attributed to LLPCs.

### *Gpi7* Mutant Vaccination Induces IgG Antibody Production Targeting Cell Wall-Localized Moonlighting Proteins of *C. albicans* in Mice

**FIGURE 4:**
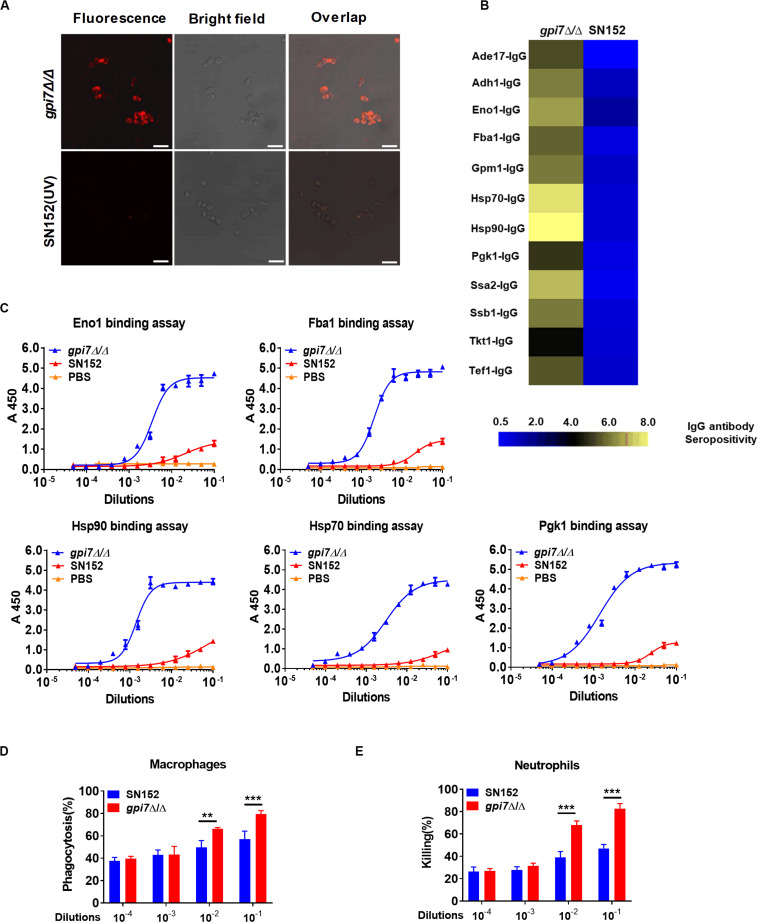
*Gpi7* mutant-vaccination induces IgG antibodies production targeting cell wall-localized moonlighting proteins of *C. albicans*. **(A)** Representative confocal microscopic analysis for IgG antibodies in *gpi7* mutant-induced antiserum from mice binding epitopes on the surface of *C. albicans* SC5314. Scale bar represents 10 μm. **(B)** Heat map of the signal intensity of IgG antibodies in *gpi7* mutant-induced antiserum binding to cell wall moonlighting proteins of *C. albicans* SC5314 was shown by Heml 1.0.3.3 (Heatmap illustrator). The IgG antibody response signals were defined as the ratio of *gpi7* mutant or parental strain SN152 induced antiserum antibody titers versus normal serum from mice. The response for each IgG antibody is color-coded, as described in the color bar. **(C)** The represent IgG antibody titers in *gpi7* mutant or parental SN152-induced antiserum were evaluated by ELISA method. **(D)** Thioglycolate-induced peritoneal macrophage phagocytosis of *C. albicans*. *C. albicans* SC5314 was co-cultured with peritoneal macrophages (MOI = 0.4) and the indicated dilution of *gpi7*-induced antiserum at 37°C for 1 h. The suspension was then plated on SDA agar for 48 h, after which *C. albicans* colonies were counted. **(E)** Thioglycolate-induced peritoneal neutrophil-mediated killing of *C. albicans*. *C. albicans* SC5314 were co-cultured with peritoneal neutrophils (MOI = 0.05) with the indicated dilution of *gpi7*-induced antiserum at 37°C for 1 h. The suspension was then plated on SDA agar for 48 h, after which *C. albicans* colonies were counted. **P < 0.01; ***P < 0.001 [(One-way ANOVA **(D,E)**].

To investigate the mechanism though which *gpi7* mutant-vaccination induced immunoprotection, we determined whether *gpi7* mutant vaccination could induce specific anti-*C. albicans* antibodies in mice. Indeed, we found that serum samples from *gpi7* mutant-vaccinated mice contained specific antibodies that recognized the surface of *C. albicans*, as demonstrated by confocal laser scanning microscopy, whereas serum samples from parental strain SN152-vaccinated mice only contained very low levels of anti-*C. albicans* antibodies (Figure 4A).

**FIGURE 5:**
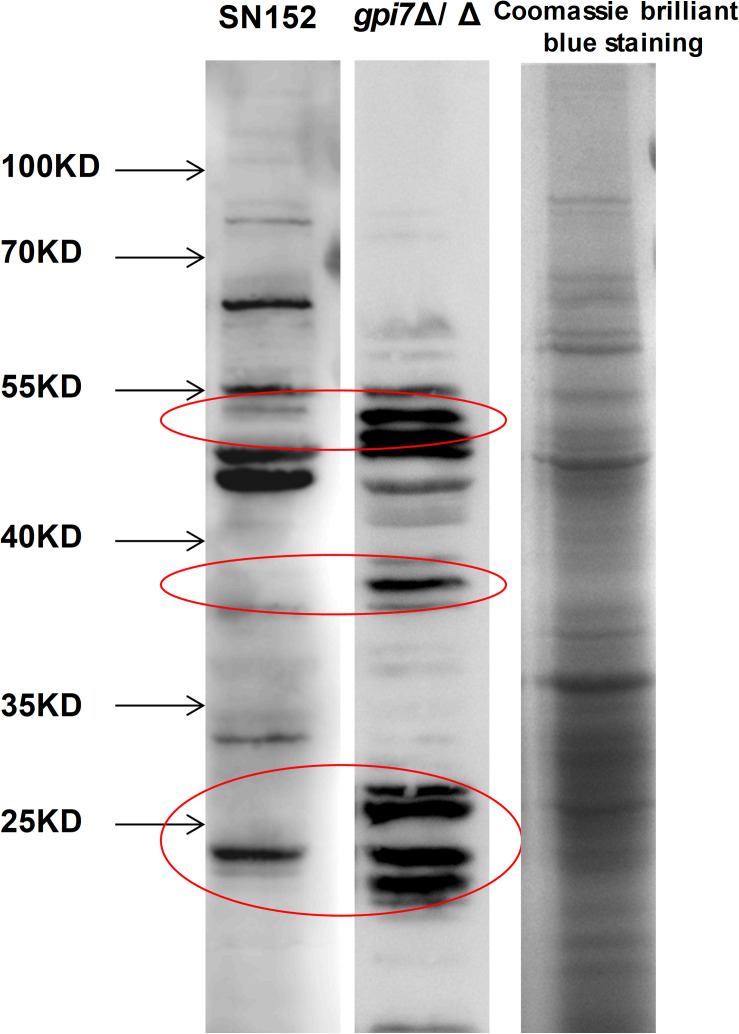
The whole CWP mixture from wild-type *C. albicans* was fracted by SDS-PAGE and then subjected to immunoblotting analysis using *gpi7* mutant or parental strain SN152-induced antiserum. Cell wall lysates from *C. albicans* SC5314 with coomassie brilliant blue staining on SDS-PAGE were used as a control. Parental strain SN152-induced antiserum detection (Band 1) or *gpi7*-induced antiserum detection (Band 2). Total CWPs from wild-type *C. albicans* fracted by SDS-PAGE and stained with Coomassie brilliant blue (Band 3).

To determine whether *gpi7* mutant-induced antibodies in mice can specifically recognize proteins from *C. albicans*, lysates from *C. albicans* SC5314 cell wall were fracted by SDS-PAGE and then subjected to immunoblotting analysis using *gpi7* mutant or parental strain SN152-induced antiserum. Cell wall lysates from *C. albicans* SC5314 stained with coomassie brilliant blue staining on SDS-PAGE were used as a control. We found that the *gpi7* mutant-induced antiserum detected proteins in cell wall lysates from *C. albicans* SC5314 were different from those detected by SN152-induced antiserum (Figure 5). LC-MS/MS analysis for the gel bands of the different proteins which isolated from the control lane identified 22 different representative cell wall proteins of *C. albicans* (Supplementary Table S3). And we found that most of these proteins were cell wall-localized moonlighting proteins, including glycolytic enzymes on the cell wall (Fba1, Pgk1, and Eno1), heat shock proteins (Hsp90, Hsp70, and the HSP70 family chaperones Ssa2 and Ssb1), oxidative stress proteins (Cat1), and general metabolic enzymes (Ade17, Adh1, Ach1, and Tkt1) (Supplementary Table S3). The results indicated that *gpi7* mutant-vaccination could protect against *C. albicans* or non-*albicans Candida* spp. caused systematic infection, suggesting that the proteins targeted by the antibodies produced after g*pi7* mutant-vaccination may share similarity among *Candida* spp. The sequence alignment results in Supplementary Figure S5 validated the high similarity of the protective antibody targeting moonlighting proteins (Eno1, Hsp90, Fba1, Hsp70, Pgk1, Ssa2, Ssb1, Ade17, Ach1).

The above-identified moonlighting proteins were produced by a bacterial expression system and were further used to investigate their IgG antibody titers in *gpi7* mutant-induced antiserum samples through ELISA. The results demonstrated that the titers of IgG antibodies targeting 12 cell wall-localized moonlighting proteins were markedly higher in *gpi7* mutant-induced antiserum samples than in those induced by SN152 strain (Figure 4B). In addition, the titers of representative IgG antibodies targeting cell wall-localized moonlighting proteins (Eno1, Fba1, Hsp90, Hsp70, and Pgk1) were shown in Figure 4C. Furthermore, we found the representative IgG antibodies could maintain higher titers until 56 days post vaccination (Supplementary Figure S6).

Neutrophils and macrophages are the primary leukocytes responsible for phagocytosis and killing of invasive fungal cells. Antibody binding to invading fungal cells can induce neutrophil or macrophage-mediated opsono-phagocytosis and opsono-killing. Serum samples from *gpi7* mutant-vaccinated mice mediated fungal opsono-phagocytosis and opsono-killing, as demonstrated by incubating *C. albicans* with macrophages and neutrophils. We found that treatment with *gpi7* mutant-induced antiserum exhibited dose-dependent opsono-phagocytosis and opsono-killing activity when macrophages and neutrophils challenged by *C. albicans* (Figures 4D,E).

### The Reactivity Differences Among Serum IgG Antibodies Targeting Cell Wall-Localized Moonlighting Proteins Are Associated With the Prognosis of IC Patients

**FIGURE 6:**
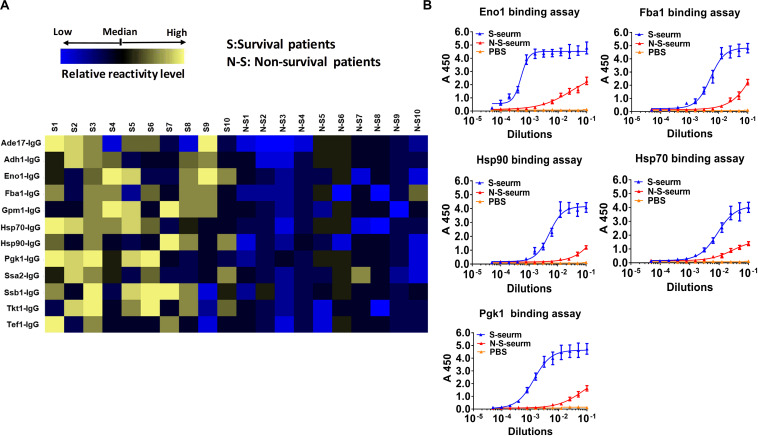
Reactivity differences among serum IgG antibodies targeting cell wall-localized moonlighting proteins of *C. albicans* are associated with the prognosis of IC patients. **(A)** Heat map of signal intensity of IgG antibodies in serum IC patients targeting cell wall-localized moonlighting proteins of *C. albicans* SC5314 was shown by Heml 1.0.3.3 (Heatmap illustrator) (*n* = 10 per group). The IgG antibody response signals were defined as the ratio of IgG antibody titers of serum from recovery IC patients or deceased IC patients versus that from healthy volunteers. The response for each IgG antibody is color-coded, as described in the color bar. **(B)** The represent IgG antibody titers in serum of recovery IC patients and deceased IC patients were evaluated by ELISA method.

Ten serum samples were collected from recovered IC patients with clinical and microbiological evidence of *C. albicans* infection, and 10 serum samples were collected from deceased IC patients. We detected the IgG antibody response targeting cell wall-localized moonlighting proteins in serum samples from IC patients. The results indicated that the titers of 12 immunodominant antibodies targeting cell wall-localized moonlighting proteins of *C. albicans* in the serum samples from recovering IC patients were markedly higher than those from the deceased IC patients (Figure 6A). The titers of the representative IgG antibodies targeting cell wall-localized moonlighting proteins (Eno1, Fba1, Hsp90, Hsp70, and Pgk1) in serum samples from the recovering IC patients were shown in Figure 6B. Meanwhile, control serum from healthy people did not show significant responsiveness to the above wall-localized moonlighting proteins (Supplementary Figure S7). These data showed that the IgG antibody response targeting cell wall-localized moonlighting proteins in the serum samples of recovering IC patients was similar to that in the *gpi7* mutant-vaccinated mice.

### T_H_2 Cell Response Is Involved in *Gpi7* Mutant Vaccination Induced Immunoprotection Against Candidiasis in Mice

**FIGURE 7:**
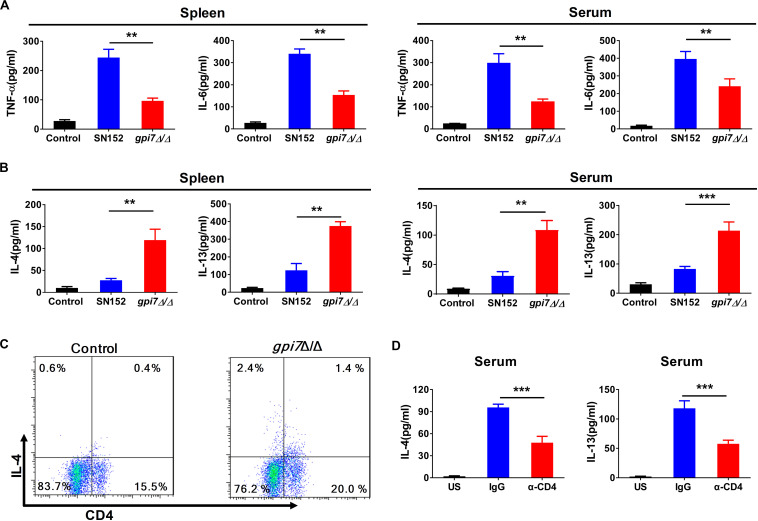
T_H_2 cell response is involved in *gpi7* mutant vaccination induced immunoprotection against candidiasis in mice. *Gpi7* mutant-vaccinated C57BL/6 mice were systemically infected with *C. albicans* SC5314 (5 × 10^5^ CFU per mouse). **(A)** ELISA results for cytokines TNF-α, IL-6 in the spleen and serum of *gpi7* mutant-vaccinated mice at day 2 post-infection (*n* = 5 per group). **(B)** ELISA results for cytokines IL-4 and IL-13 in the spleen and serum of *gpi7* mutant-vaccinated mice at day 2 post-infection (*n* = 5 per group). **(C)** Flow cytometry analysis for CD4^+^IL-4^+^ T cells in the spleen of *gpi7* mutant-vaccinated mice at day 2 post-infection. Data are representative images of 5 mice. **(D)** IL-4 and IL-13 levels in *gpi7* mutant-vaccinated mice given intravenous injection of control IgG and neutralizing anti-CD4 antibody (200 μg per mouse) twice (*n* = 5 per group). **P < 0.01; ***P < 0.001 (Non-parametric one-way ANOVA).

Cytokines play crucial roles in the initiation and amplification of B cell-mediated humoral immunity (Paul and Zhu, 2010). Our results indicated that the levels of T_H_1-type effector cytokines, such as tumor necrosis factor (TNF)-α and IL-6, in the spleen and serum of *gpi7* mutant-vaccinated mice after challenged by *C. albicans* SC5314 were significantly lower than those in the spleen and serum of the parental strain SN152-vaccinated mice (Figure 7A). However, we found that *gpi7* mutant vaccination could significantly increase the levels of T_H_2-type effector cytokines, such as IL-4 and IL-13, in the spleen and serum of mice when challenged by *C. albicans* SC5314, which suggests that these play a role in the immunoprotection against candidiasis (Figure 7B).

CD4^+^T cells are important sources of T_H_2-type effector cytokines. The analysis of CD4^+^ lymphocytes revealed a significantly greater frequency of IL-4 producing CD4^+^ lymphocytes in the spleen of *gpi7* mutant-vaccinated mice than the parental strain SN152-vaccinated mice (Figure 7C). However, we observed a similar frequency of IL-17A-producing CD4^+^T cells and IFN-γ producing CD4^+^T cells (Supplementary Figure S5). Depletion CD4^+^ T cells in *gpi7* mutant-vaccinated mice by a neutralizing anti-CD4 antibody prior to challenge with *C. albicans* SC5314 significantly reduced the concentrations of IL-4 and IL-13 in the serum of *gpi7* mutant-vaccinated mice (Figure 7D). These data indicated that CD4^+^ T_H_2 cells mediated immune responses were involved in the immunoprotection conferred by *gpi7* mutant vaccination.

### Dectin-1 Mediated IL-18 Production Is Required for the Generation of Candidacidal Antibodies in *Gpi7* Mutant-Vaccinated Mice

**FIGURE 8:**
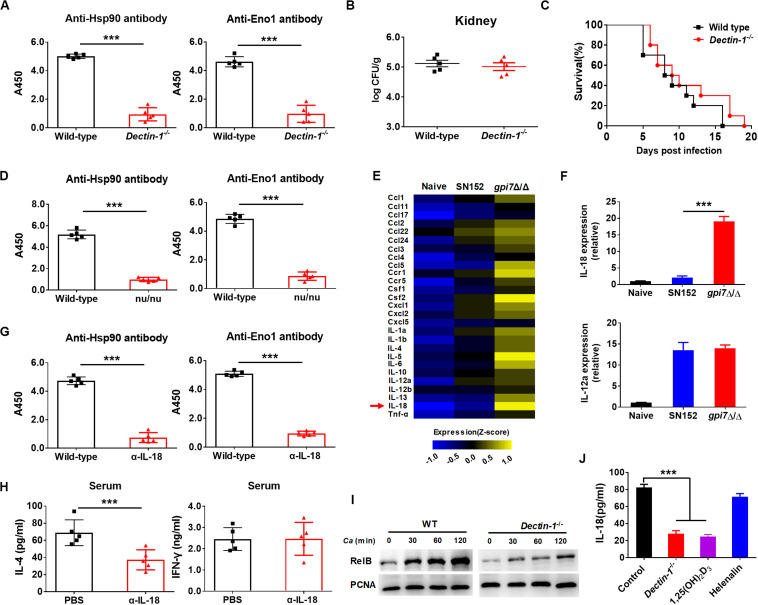
*Gpi7* mutant induced antibodies generation in mice was Dectin-1 dependent and relied on IL-18. **(A)** Anti-Hsp90 and Anti-Eno1 antibodies assay by ELISA in *gpi7* mutant-vaccinated wild-type C57BL/6 mice or Dectin-1-deficient C57BL/6 mice (*n* = 5 per group). **(B,C)** *Gpi7* mutant-vaccinated wild-type C57BL/6 mice or Dectin-1-deficient C57BL/6 mice were systemically infected with *C. albicans* SC5314 (5 × 10^5^ CFU per mouse). The kidney fungal burden was determined at day 2 post-infection (**B**, *n* = 5 per group), and the survival was monitored for 20 days’ post-infection (**C**, *n* = 10 per group). **(D)** Anti-Hsp90 and Anti-Eno1 antibodies assay by ELISA in *gpi7* mutant-vaccinated wild-type BALB/c mice or athymic nude mice (BALB/c background) (n = 5 per group). **(E)** Peritoneal macrophages were stimulated with *gpi7* mutant for 24 h and blank medium was used as a control. Total RNA was extracted, and real-time RT-PCR assays on inflammatory cytokines was performed. Data shown are heat maps for assayed gene panels. Arrows highlight the upregulated IL-18. **(F)** Real-time RT-PCR analysis of IL-18 and IL-12a expression in peritoneal macrophages as in **(E)**, results were normalized to those of the control gene Gapdh. **(G)** Anti-Hsp90 and Anti-Eno1 antibodies assay by ELISA in *gpi7* mutant-vaccinated C57BL/6 mice given intravenous injection of control IgG and neutralizing anti-IL-18 antibody (100 μg per mouse) twice (n = 5 per group). **(H)** ELISA results for cytokines IL-4 and IFN-γ in serum of mice as in **(G)** challenged by *C. albicans* (5 × 10^5^ CFU per mouse, n = 5 per group). **(I)** Immunoblot analysis of RelB in the nucleus of peritoneal macrophages isolated from wild-type mice (Dectin-1^+^*^/^*^+^) and Dectin-1-deficient mice (Dectin-1^– /–^) and assessed at 0–2 h after challenge with gpi7 mutant (MOI = 5). **(J)** Peritoneal macrophages were pretreated with P65 inhibitor helenalin (20 μmol/L), RelB inhibitor 1,25(OH)_2_D_3_ (0.1 μmol/L) or PBS, and then stimulated with *gpi7* mutant. The production of IL-18 was determined by ELISA. ***P < 0.001 [Non-parametric *t*-test **(A,B,D,G,H)**; Log-rank test **(C)**; One-way ANOVA **(F,J)**].

Dectin-1 engagement by fungal β-(1,3)-glucan activates innate immune cells to respond and renders antigen-presenting cells competent to prime T cells differentiation (Rogers et al., 2005; Taylor et al., 2007). Our previous study indicated that β-(1,3)-glucan on the surface of *gpi7* mutant was exposed and could engage host Dectin-1 (Shen et al., 2015). Therefore, we investigated whether Dectin-1 engagement by β-(1,3)-glucan involved in the immunoprotection of *gpi7* mutant-vaccinated mice. We vaccinated Dectin-1-deficient mice with the *gpi7*-mutant strain and found that *gpi7* mutant vaccination could not induce protective antibodies such as anti-Hsp90 and anti-Eno1 antibodies (Figure 8A). In addition, vaccination of Dectin-1-deficient mice with the *gpi7*-mutant strain could not protect against lethal invasive *C. albicans* infection, as indicated by fungal burdens in the kidneys and survival time (Figures 8B,C). Experiments with athymic nude mice found that *gpi7* mutant vaccination also could not induce the generation of protective antibodies such as anti-Hsp90 and anti-Eno1 antibodies (Figure 8D). Therefore, we hypothesized that the T_H_2 cell-mediated protective effect induced by the *gpi7*-mutant strain is dependent on Dectin-1.

Dectin-1 receptor triggers innate immune cells to secret cytokines to prime naive CD4^+^ T cells differentiation. Therefore, a real-time RT-PCR assay was performed on peritoneal macrophages challenged by *gpi7* mutant or parental SN152 strain to identify the potential cytokines initiation naive CD4^+^ T cell differentiation. Compared to other inflammatory related cytokines, we found that peritoneal macrophages challenged by *gpi7* mutant had relative higher expression of the gene encoding IL-18 than those challenged by parent strain SN152 (Figures 8E,F). In addition, neutralization of endogenous IL-18 significantly reduced protective antibodies generation and serum concentration of IL-4 in *gpi7* mutant-vaccinated mice after challenged by *C. albicans* (Figures 8G,H). However, neutralization of IL-18 had no significant influence on the serum concentration of IFN-γ (Figure 8H). Thus, we hypothesized that β-(1,3)-glucan on the surface of the *gpi7* mutant activated the Dectin-1 receptor to facilitate the secretion of IL-18 from host innate immune cells, and then induced type 2 responses in *gpi7* mutant-vaccinated mice.

Dectin-1 induces cytokines expression requires NF-κB signaling. Our previous studies indicated that *gpi7* mutant could activate classical NF-κB (p65) by Dectin-1 receptor in macrophages (Shen et al., 2015). Here we show that *gpi7* mutant also facilitated nuclear translocation of non-canonical NF-κB subunit RelB in a Dectin-1 dependent manner (Figure 8I). Furthermore, we found that RelB inhibitor 1,25-dihydroxyvitamin D3 [1,25(OH)_2_D_3_] (Mineva et al., 2009) and Dectin-1 deficiency could markedly impair the *gpi7* mutant-induced production of IL-18 in peritoneal macrophages (Figure 8J). However, P65 inhibitor helenalin (Widen et al., 2017) had no significant influence on IL-18 production (Figure 8J). Thus, these data suggested that the Dectin-1 dependent activation of non-canonical NF-κB subunit RelB is required for the *gpi7* mutant-induced priming of IL-18 production.

## Discussion

Invasive infections caused by *Candida* spp. represents the fourth leading cause of healthcare-associated infections (Arendrup, 2010; Kullberg and Arendrup, 2015; McCarty and Pappas, 2016; Yang et al., 2017). The limited ability of antifungal therapy renewed the interest for effective vaccines research. In this study, we reported that the avirulent *gpi7* mutant *C. albicans* could induce generation of candidacidal antibodies. We found that the generation of protective antibodies in *gpi7* mutant-vaccinated mice was dependent on Dectin-1 and that IL-18 plays a central role in this process. Mechanistically, we showed that Dectin-1 engagement by *gpi7* mutant was associated with translocation of non-canonical NF-κB subunit RelB to nucleus for facilitating IL-18 production. Our study not only suggests a new active vaccination strategy for *Candida* spp. infection but also provides evidence showing that the Dectin-1 receptor facilitates IL-18 production for the host generation of candidacidal antibodies.

Under normal conditions, *C. albicans* can shield cell surface β-(1,3)-glucan to avoid host phagocyte Dectin-1 recognition (Gantner et al., 2005; Wheeler et al., 2008). In the previous study, we constructed a *gpi7* mutant *C. albicans* strain with surface β-(1,3)-glucan exposure, and this mutant could stimulate Dectin-1 receptor to drive T_H_1 and T_H_17 cell mediated immune responses (Shen et al., 2015). In this study, we found the *gpi7* mutant could also elicit type 2 responses with candidacidal antibody generation and protect mice from lethal *Candida* spp. infection, including those caused by *C. albicans* and non-*albicans Candida* spp. (*C. glabrata*, *C. krusei*, *C. parapsilosis* and *C. tropicalis*) (Figures 1, 2, 4). However, other avirulent *C. albicans* mutant without surface β-(1,3)-glucan exposure could not confer immunoprotection against wild-type strain reinfection, suggesting the central role of β-(1,3)-glucan (Supplementary Figure S2). In addition to GPI-CWPs, a certain number of moonlighting proteins, which are multifunctional proteins participating in unrelated biological processes and are not the result of gene fusion, have been characterized in the cell wall of *C. albicans* (Gancedo et al., 2016; Serrano-Fujarte et al., 2016). We found that the main IgG antibodies in *gpi7* mutant-induced antiserum were targeted to moonlighting proteins on cell wall of *C. albicans* (Figure 4). Some studies have reported that the moonlighting proteins of *Candida* spp. can induce IgG antibodies generation in host and protect against fungal reinfection (Pachl et al., 2006; Clancy et al., 2008; Xin et al., 2008; Li et al., 2011; Masek et al., 2011; Xin and Cutler, 2011). Furthermore, we found that the high titer of IgG antibody was maintained for a long time after *gpi7* mutant-vaccination, which suggests the longevity of the protective effects of this vaccination against IC (Supplementary Figure S6). Previous studies have been demonstrated that purified glucan could enhance innate antifungal immune response (Quintin et al., 2012; Cheng et al., 2014) and glucan-based vaccine could increase resistance of experimental animal to invasive candidiasis (Bromuro et al., 2010; Liao et al., 2015, 2016). Heat inactivated cell wall β-glucan of *C. albicans* was exposed, but the protein on the cell wall surface was denatured. Our results indicated that heat inactivated *C. albicans*-vaccination and fungal glucan-vaccination did not increase resistance of mice to invasive candidiasis by decreasing fungal burden or improving survival in our study (Supplementary Figure S9). The above results further confirmed the protection of *gpi7* mutant-vaccination is mainly mediated by immunity to *Candida* moonlighting proteins. At last, we further determined that the IgG profiles in serum from recovering IC patients were similar to those of *gpi7* mutant-vaccinated mice, which suggests that antibodies that target moonlighting proteins favor the prognosis of IC (Figure 6). However, patients with invasive candidiasis often suffer from multiple co-morbidities along with immunosuppression, which may influence the antibodies response targeting moonlighting proteins. In addition, the antibodies response to *candida* moonlighting proteins in the patients with other forms of candidiasis, such as oral thrush and vaginal candidiasis, deserves further study in the future.

Host innate immune receptors can sense pathogens and then trigger an effective adaptive immunity. Our results indicated that *gpi7* mutant induced protective effect and that antibody generation was depended on Dectin-1 receptor. Dectin-1 triggers Syk kinase-dependent inflammatory cytokines secretion to induce antifungal immunity (Taylor et al., 2007). We found that both the immunoprotection and antibody generation induced by the *gpi7* mutant were dependent on Dectin-1 (Figures 8A–C), suggesting a new linkage between Dectin-1 receptor and adaptive humoral immunity. IL-18 was originally identified as a factor that enhances IFN-γ production by T_H_1 cells, particularly in association with IL-12 (Wawrocki et al., 2016; Kaplanski, 2018; Nakanishi, 2018). Thus, IL-18 is regarded as a proinflammatory cytokine that primes type 1 responses, but whether IL-18 can trigger type the 2 response to facilitate candidacidal antibodies generation is unclear. IL-18 is synthesized as an inactive precursor and is induced by NF-κB pathway (Wawrocki et al., 2016; Nakanishi, 2018). Nlrp3 inflammasome orchestrating Caspase-1 activation is essential for the cleavage of pro-IL-18 into their mature and biologically active forms, and plays a role in host antifungal immune response (Thomas et al., 2009; Karki et al., 2015; Kasper et al., 2018; de Castro-Jorge et al., 2019; Swidergall et al., 2019). In addition, recent study revealed that IL-18 production is essential for host defense against fungus *Paracoccidioides brasiliensis* (Ketelut-Carneiro et al., 2015). In this study, we found that IL-18 expression in peritoneal macrophages challenged with the *gpi7* mutant was significantly increased in a Dectin-1-dependent manner (Figures 8F,J). More notably, neutralization of endogenous IL-18 significantly reduced protective antibodies generation in *gpi7* mutant-vaccinated mice (Figure 8G). Therefore, Dectin-1 dependent IL-18 secretion represents the key factor for the type 2 responses with candidacidal antibodies generation in the present study.

The NF-κB transcription factors consist of five members: p52 and its precursor (p100), RelB, p50 and its precursor (p105), p65, and c-Rel (Hayden and Ghosh, 2004; Mitchell et al., 2016). Dectin-1 activates not only the Syk-dependent canonical NF-κB subunits p65 and c-Rel, but also the non-canonical NF-κB subunit RelB (Gringhuis et al., 2009; Xu et al., 2018). p65 and c-Rel activation induced by Dectin-1 can prime cytokines IL-12, IL-6 and IL-1β production and drive the T_H_1 and T_H_17 cells mediated responses (Gross et al., 2006; Yang et al., 2011). While orchestration of RelB by Dectin-1 often initiates the secretion of cytokines IL-4 and IL-5 to drive T_H_2 cell mediated response (Gringhuis et al., 2009; Xu et al., 2018). Our previous study indicated *gpi7* mutant could activate canonical NF-κB subunits p65 through Dectin-1 receptor and thereby prime T_H_1 and T_H_17 cell responses against fungal infections (Shen et al., 2015). In this study, we found *gpi7* mutant also facilitated nuclear translocation of non-canonical NF-κB subunit RelB through activating Dectin-1 receptor (Figure 8I). More notably, we found that the *gpi7* mutant induced IL-18 production in peritoneal macrophages also depended on RelB activation, but not p65 (Figure 8J). Therefore, our results suggested that Dectin-1 mediated RelB activation could regulated IL-18 production and then prime candidacidal antibodies generation.

In conclusion, our present study revealed that vaccination with the avirulent *C. albicans gpi7* mutant could protect against invasive *Candida* infection, and thus provides a new active vaccination strategy. The immunoprotection attributed to type 2 immune responses which mediated generation of candidacidal antibodies. We further demonstrated that Dectin-1 engagement by the cell surface β-1,3-glucan of *gpi7* mutant could trigger RelB activation to prime IL-18 expression and subsequently regulate the generation of candidacidal antibodies, and these findings establish a new paradigm regarding the link between the Dectin-1 mediated innate immune response and adaptive humoral immunity.

## Data Availability Statement

All datasets generated for this study are included in the article/Supplementary Material.

## Ethics Statement

The studies involving human participants were reviewed and approved by Institutional Ethical Board of Shanghai Tenth People’s Hospital. The patients/participants provided their written informed consent to participate in this study. The animal study was reviewed and approved by Institutional Animal Care and Use Committee of Tongji University. Written informed consent was obtained from the owners for the participation of their animals in this study.

## Author Contributions

M-MA, ZZ, Y-YJ, and HS conceptualized the study. HS and S-MC performed the experiments on vaccination with *gpi7* mutant protecting mice from candidiasis, and wrote the manuscript. J-JS, WF, and S-YG performed the vaccination experiments. Y-LC and Y-DW performed flow cytometry analysis. W-TH, X-RQ, and YZ performed analysis on IgG antibodies in serum of *gpi7* mutant-vaccinated mice. X-YH and LL performed confocal laser scanning microscopy observation. YY performed the analysis of IgG antibodies in serum of IC patients, and wrote the manuscript. M-MA, ZZ, HS, and Y-YJ supervised the study and wrote the manuscript. All authors contributed to the article and approved the submitted version.

## Conflict of Interest

The authors declare that the research was conducted in the absence of any commercial or financial relationships that could be construed as a potential conflict of interest.
