# Supplementary material for: Dectin-1 Facilitates IL-18 Production for the Generation of Protective Antibodies Against Candida albicans
Source: Front Microbiol. 2020 Jul 16;11:1648. doi: 10.3389/fmicb.2020.01648 (PMC7378971; doi:10.3389/fmicb.2020.01648)
Supplement: Supplementary file 1 [file Data_Sheet_1.DOCX]

**FIGURE S1 |** The kidney **(A)** and liver fungal burden **(B)** of *gpi7* mutant-vaccinated and parental *C. albicans* SN152-vaccinated C57BL/6 mice at day 7 post systemically infected with *C. albicans* SC5314 (5×10^5^ CFU per mouse). Data are representative of three independent experiments. ***, *P* < 0.001 (Nonparametric *t*-test).

**

**

**FIGURE S2 |** Vaccination with avirulent *SSA1* mutant could not confer resistance to reinfection by wild-type *C. albicans*. C57BL/6 mice were intravenously vaccinated with live *ssa1* mutant and its complemental strain (UV-inactive) (5×10^5^ CFU per mouse) at day 1 and day 14, and re-infected with wild-type *C. albicans* SC5314 (5×10^5^ CFU per mouse) at day 28. The kidney fungal burden was determined at day 2 post-infection.

**

**

**FIGURE S3 |** ELISA assays for IFN-γ and IL-17 in serum of g*pi7* mutant-vaccinated mice with anti-IFN-γ antibody (A) and anti-IL-17A antibody treatment (B). *Gpi7* mutant-vaccinated C57BL/6 mice were treated with anti-IFN-γ antibody (500 μg per mouse) **(A)** or anti-IL- 17A (100 μg per mouse) **(B)**. And the control mice received same volume of PBS buffer (n=5). ND, Not detectable.





**FIGURE S4 |** Flow cytometry for B220^+^CD273^+^CD80^+^CD73^+^ B_mem_ in the spleen of *gpi7* mutant-vaccinated and parental strain SN152-vaccinated C57BL/6 mice. Data are representative images of five mice.





**FIGURE S5 |** Similarities of moonlighting proteins targeted by the protective antibodies elicited by *gpi7*-mutant among *Candida* spp. including *C. glabrata*, *C. parapsilosis*, *C. krusei* and *C. tropicalis* and *C. albicans*. Similarities of *C. albicans* moonlighting proteins were regarded as 100 percent. (A) Eno1. (B) Hsp90. (C) Fba1. (D) Hsp70. (E) Pgk1. (F) Ssa2. (G) Ssb1. (H) Ade17. (I) Ach1.


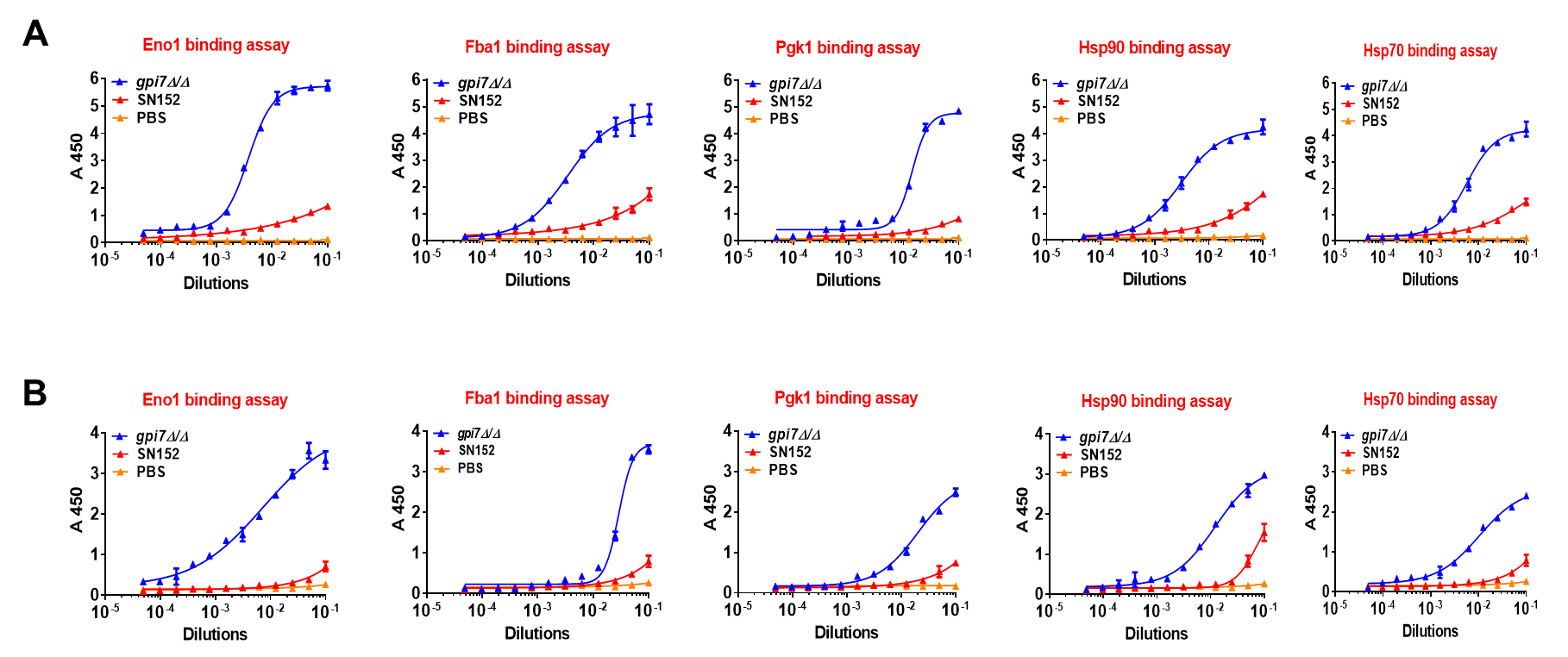


**FIGURE S6 |** The represent IgG antibody titers in serum of gpi7 mutant mutant-vaccinated and parental strain SN152-vaccinated C57BL/6 mice on 42 day **(A)** and 56 day **(B)** post first immunity were evaluated by ELISA method.





**FIGURE S7 |** Heat map of signal intensity of IgG antibodies in serum healthy (n=10 per group) individual targeting cell wall-localized moonlighting proteins of *C. albicans* SC5314 was shown by Heml 1.0.3.3 (Heatmap illustrator).





**FIGURE S8 |** Flow cytometry analysis for CD4^+^IL-17^+^ T cells **(A)** and CD4^+^IFN-γ^+^ T cells **(B)** in the spleen of *gpi7* mutant-vaccinated mice at day 2 post systemically infected with *C. albicans* SC5314. Data are representative images of five mice.

**

**

**FIGURE S9 |** Comparation of the protective effect of *gpi7* mutant-vaccination, purified fungal glucan (zymosan)-vaccination and heat inactivated (HI) *C. albicans* SN152-vaccination. C57BL/6 mice were intravenously vaccinated at day 1 and day 14, and re-infected with wild-type *C. albicans* SC5314 at day 28. The survival was surveyed for 40 days’ post-infection (**A**). The kidney (**B**) and liver (**C**) fungal burden was determined at day 2 post-infection. ***, *P* < 0.001 [Log-rank test **(A);** Nonparametric one-way ANOVA **(B, C)**].

**TABLE S1 |** **Primers used for amplifying the gene encoding *C. albicans* proteins.**

| **Primers used for amplifying the gene encoding  *C. albicans* proteins** |
| --- |
| Eno1-F: GGATCCATGTCTTACGCCACTAAAA  Eno1-R: CTCGAGCTTACAATTGAGAAGCCTTT |
| Hsp90-F: GGATCCATGGCTGACGCAAAAGTT  Hsp90-R: CTCGAGTAATCAACTTCTTCCA |
| Fba1-F: GGATCCATGGCTCCTCCAGCAGT  Fba1-R: CTCGAGTTACAATTGTCCTTTAGT |
| Hsp70-F: GGATCC ATGTCTAAAGCTGTTGG  Hsp70-F: CTCGAGTTAATCAACTTCTTCAAC |
| Pgk1-F: GGATCCATGTCATTATCTAACAAAT  Pgk1-R: CTCGAGTTAGTTTTTGTTGGAAA |
| Gpm1-F: GGATCCATGCCAAAGTTAGTTTT  Gpm1-R: CTCGAGGCTCAAGGTCAAAAGAA |
| Ade17-F: GGATCCATGTCCGACAAACAACAC  Ade17-R: CTCGAGCTAATGATGGAACAAAC |
| Ssa2-F: GGATCCATGTCTAAAGCTGTTGGTA  Ssa2-R: CTCGAGTGTTGAAGAAGTTGATTA |
| Ssb1-F: GGATCCATGGCTGACGGTGTTTTC  Ssb1-R: CTCGAGTTAACGGGTAGCCATAC |
| Adh1-F: GGATCCATGTCTGAACAAATCC  Adh1-R: CTCGAGTTATTTACTGGTGTCCAA |
| Tkl1-F: GGATCCATGCCTTCTCTTGATGAA  Tkl1-R: CTCGAGTTAGAAAGCTCTGTCTAA |
| Tef-1-F: GGATCCATGGGTAAAGAAAAAACT  Tef-1-R: CTCGAGTTATTTCTTAGCAGCTTTT |

**TABLE S2 |** **Primers used for quantitative real-time PCR**

| **Primers used for quantitative real-time PCR** |
| --- |
| CCL1-F: CCCAGCTGTGGTATTCAGGC  CCL1-R: GTGATTTTGAACCCACGTTTTG |
| CCL11-F: CCAGGCTCCATCCCAACTT  CCL11-R: TGGTGATTCTTTTGTAGCTCTTCAGT |
| CCL17-F: GGATGCCATCGTGTTTCTGA  CCL17-R: GCCTTCTTCACATGTTTGTCTTTG |
| CCL2-F: GCTGGAGCATCCACGTGTT  CCL2-R: ATCTTGCTGGTGAATGAGTAGCA |
| CCL22-F: TGCCAGGACTACATCCGTCA  CCL22-R: GGCAGGATTTTGAGGTCCAG |
| CCL24-F: TGGTAGCCTGCGCGTGTT  CCL24-R: AAGGACGTGCAGCAAGATGA |
| CCL3-F: CCAAGTCTTCTCAGCGCCAT  CCL3-R: GAATCTTCCGGCTGTAGGAGAAG |
| CCL4-F: TCTGCGTGTCTGCCCTCTC  CCL4-R: TGCTGAGAACCCTGGAGCA |
| CCL5-F: GCAAGTGCTCCAATCTTGCA  CCL5-R: CTTCTCTGGGTTGGCACACA |
| CCR1-F: TTAGCTTCCATGCCTGCCTTATA  CCR1-R: TCCACTGCTTCAGGCTCTTGT |
| CCR5-F: CAAGACAATCCTGATCGTGCAA  CCR5-R: TCCTACTCCCAAGCTGCATAGAA |
| CSF1-F: TTGCCAAGGAGGTGTCAGAACACT  CSF1-R: AAGGCAATCTGGCATGAAGTCTCC |
| CSF2-F: TGTTGGCCAAGCACTATGAG  CSF2-R: CAAAGGGGATGGTGAAAAGA |
| CXCL1-F: ATCCAGAGCTTGAAGGTGTTG  CXCL1-R: GTCTGTCTTCTTTCTCCGTTACTT |
| CXCL2-F: ATGCCTGAAGACCCTGCCAAG  CXCL2-R: GGTCAGTTAGCCTTGCCTTTG |
| CXCL5-F: ACAGTGCCCTACGGTGGAAGT  CXCL5-R: CGAGTGCATTCCGCTTAGCTT |
| IL-1a-F: ATGGCCAAAGTTCCTGACTTGTTT  IL-1a-R: CCTTCAGCAACACGGGCTGGTC |
| IL-1β-F: TGTGCAAGTGTCTGAAGCAGC  IL-1β-R: TGGAAGCAGCCCTTCATCTT |
| IL-4-F: ACTTGAGAGAGATCATCGGCA  IL-4-R: AGCTCCATGAGAACACTAGAGTT |
| IL-5-F: AGGATGCTTCTGCACTTGAG  IL-5-R: TCTGTACTCATCACACCAAG |
| IL-6-F: ACTTCCATCCAGTTGCCTTC  IL-6-R:GTCTCCTCTCCGGACTTGTG |
| IL-10-F: TGCCTTCAGTCAAGTGAAGAC  IL-10-R: AAACTCATTCATGGCCTTGTA |
| IL-12a-F: cagcatgtgtcaatcacgctac  IL-12a-R: tgtggtcttcagcaggtttc |
| IL-12b-F: AGACATGGAGTCATAGGCTCTG  IL-12b-R: CCATTTTCCTTCTTGTGGAGCA |
| IL-13-F: GCAGTCCTGGCTCTTGCTTG  IL-13-F: TGCTTTGTGTAGCTGAGCAG |
| IL-18-F: GACTCTTGCGTCAACTTCAAGG  IL-18-R: CAGGCTGTCTTTTGTCAACGA |
| TNF-α: GGCGGTGCCTATGTCTCAG  TNF-α: GGGCAGCCTTGTCCCTTGA |
| GAPDH-F: AAGAAGGTGGTGAAGCAGGC  GAPDH-R: TCCACCACCCAGTTGCTGTA |

**Table S3. Specific proteins from *C. albicans* detecting by *gpi7*-induced antibodies in immunized serum**

| Protein name | Mr (kDa) | PI | Matching  peptides | Accession  number | Description |
| --- | --- | --- | --- | --- | --- |
| **Hsp90** | **80.8** | **4.81** | **32** | **P46598** | Essential chaperone, regulates several signal transduction pathways and temperature-induced morphogenesis; activated by heat shock, stress; localizes to surface of hyphae, not yeast cells. |
| **Tkt1** | **73.8** | **5.53** | **21** | **O94039** | Putative transketolase; localizes to surface of yeast cells，antigenic in human or murine infection; possibly essential. |
| **Hsp70** | **70.3** | **5.05** | **18** | **P41797** | Putative hsp70 chaperone; role in entry into host cells; heat-shock, amphotericin B, cadmium, ketoconazole-induced; surface localized in yeast and hyphae; antigenic in host. |
| **Ssa2** | **70.1** | **4.95** | **21** | **P46587** | HSP70 family chaperone; cell wall fractions; antigenic; beta-defensin peptides impport; ATPase domain binds histatin 5; at hyphal surface, not yeast. |
| **Ssb1** | **66.5** | **5.25** | **20** | **Q5A397** | HSP70 family heat shock protein; mRNA in yeast and germ tubes; at yeast cell surface, not hyphae; antigenic in human/mouse infection; macrophage |
| **Ade17** | **64.9** | **6.11** | **25** | **Q5A6R2** | 5-Aminoimidazole-4-carboxamide ribotide transformylase, enzyme of adenine biosynthesis; antigenic in human; soluble protein in hyphae; not induced during GCN response |
| **Ach1** | **58.0** | **6.42** | **14** | **P83773** | Acetyl-coA hydrolase; acetate utilization; nonessential; soluble protein in hyphae; antigenic in human; induced on polystyrene adherence; farnesol-, ketoconazole-induced; no human or murine homolog; stationary phase-enriched protein |
| **Atp2** | **55.8** | **4.94** | **22** | **Q6CFT7** | F1 beta subunit of F1F0 ATPase complex; antigenic in human, mice; induced by ciclopirox olamine; caspofungin repressed; macrophage/pseudohyphal-induced |
| **Cdc19** | 55.4 | **6.54** | **17** | **P46614** | Pyruvate kinase at yeast cell surface; Gcn4/Hog1/GlcNAc regulated; Hap43/polystyrene adherence induced; repressed by phagocytosis/farnesol; hyphal growth role; stationary phase enriched; flow model biofilm induced; Spider biofilm repressed |
| **Cat1p** | **54.9** | **6.18** | **15** | **O13289** | Catalase; resistance to oxidative stress, neutrophils, peroxide; role in virulence; regulated by iron, ciclopirox, fluconazole, carbon source, pH, Rim101, Ssn6, Hog1, Hap43, Sfu1, Sef1, farnesol, core stress response; Spider biofilm induced |
| **Cys4** | **53.9** | **6.02** | **7** | **Q59T95** | RAM cell wall integrity signaling network protein; cell separation, azole sensitivity; needed for hyphal growth; insertion mutation near 3' end of gene increases caspofungin sensitivity |
| **Atp1** | **52.9** | **8.49** | **23** | **Q59TB2** | ATP synthase alpha subunit; antigenic in human/mouse; at hyphal surface; ciclopirox, ketoconazole, flucytosine induced; Efg1, caspofungin repressed; |
| **Cit1** | **52.0** | **6.95** | **12** | **A0A1D8PSH3** | Citrate synthase; induced by phagocytosis; induced in high iron; Hog1-repressed; Efg1-regulated under yeast, not hyphal growth conditions; present in exponential and stationary phase; Spider biofilm repressed; rat catheter biofilm induced. |
| **Tef1** | **50.0** | **9.14** | **10** | **P0CY35** | Translation elongation factor 1-alpha; at cell surface; binds human plasminogen; macrophage/pseudohyphal-induced; induced in RHE model of oral candidiasis, in clinical oral candidiasis isolates; possibly essential; Spider biofilm repressed. |
| **Eno1** | **48.0** | **5.54** | **17** | **P30575** | Enolase, involved in glycolysis and gluconeogenesis; also has transglutaminase activity involved in assembly of cell wall polysaccharides; major cell-surface antigen; binds host plasmin/plasminogen; immunoprotective. |
| **Pgk1** | **45.3** | **6.07** | **24** | **P46273** | Phosphoglycerate kinase; localizes to cell wall and cytoplasm; antigenic in murine/human infection; flow model biofilm, Hog1-, Hap43-, GCN-induced; repressed upon phagocytosis. |
| **Fba1** | **39.2** | **5.69** | **15** | **Q9URB4** | Fructose-bisphosphate aldolase; glycolytic enzyme; antigenic in murine/human infection; regulated by yeast-hypha switch; induced by Efg1, Gcn4, Hog1, fluconazole; phagocytosis-repressed; flow model biofilm induced; Spider biofilm repressed. |
| **Xyl2** | **38.8** | **5.62** | **14** | **A0A1D8PUB4** | D-xylulose reductase; immunogenic in mice; soluble protein in hyphae; induced by caspofungin, fluconazole, Hog1 and during cell wall regeneration; Mnl1-induced in weak acid stress. |
| **Adh1** | **36.9** | **5.85** | **12** | **A0A1D8PP43** | Alcohol dehydrogenase; oxidizes ethanol to acetaldehyde; at yeast cell surface; immunogenic in humans/mice; complements S. cerevisiae adh1 adh2 adh3 mutant; fluconazole, farnesol-induced; flow model biofilm induced; Spider biofilm repressed. |
| **Tdh1** | **35.8** | **6.61** | **10** | **Q92211** | NAD-linked glyceraldehyde-3-phosphate dehydrogenase; binds fibronectin, laminin; at cell surface; antigenic in infection; farnesol-repressed; stationary phase-enriched; GlcNAc-induced; flow model biofilm induced; Spider biofilm repressed. |
| **Yst1** | **28.7** | **4.80** | **12** | **O42817** | Ribosome-associated protein; antigenic in mice; complements S. cerevisiae yst1 yst2 mutant; similar to laminin receptor; predicted S/T phosphorylation, N-glycosylation, myristoylation, Hap43-, Gcn4-regulated; Spider biofilm repressed |
| **Gpm1** | **27.4** | **5.79** | **13** | **P82612** | Phosphoglycerate mutase; surface protein that binds host complement Factor H and FHL-1; antigenic; fluconazole, or amino acid starvation (3-AT) induced, farnesol-repressed; Hap43, flow model biofilm induced; Spider biofilm repressed |
